# Out With the Old; in With the New

**DOI:** 10.5812/kowsar.22517464.2642

**Published:** 2012-01-15

**Authors:** Mohammad Hosein Kalantar Motamedi

**Affiliations:** 1 Trauma Research Center, Baqiyatallah University of Medical Sciences, Tehran, IR Iran

Rapid advancements and research in the fields of medicine and the growing number of specialists are narrowing the spectrum of healthcare that general practitioners (GPs) can provide. In order to provide patients with an accurate diagnosis and proper treatment care, GPs have to refer their patients to the relevant specialist. This is why more and more GPs are inclined to pursue a specialty and more specialists are inclined to pursue a subspecialty. This trend as well as the daily increase of medical doctors is why fewer patients refer to GPs. This is the primary basis for upgrading journals of general medicine to more specialized ones. Having said that, it is with great pride that I introduce the initiation of the journal **Trauma Monthly (TM)** formerly known as the **Kowsar Medical Journal (KMJ)**. KMJ was a persian medical journal targeting GPs as the primary readers and was not international; it failed to provide a worldwide forum to address issues of a specific field. In accord with the new policy adopted by our ministry of health, KMJ was upgraded to TM. This necessitated a total revamp of the journal with new editors, consultants and an international editorial board. This process was a time-taking, pain-staking undertaking by which the new English Medical journal TM evolved.

TM seeks to provide scientists, specialists and traumatologists a medium to publish contemporary research in the fields of Trauma, Surgery and Emergency Medicine. It is an international peer-reviewed medical journal indexed in Scopus and Embase. TM aims to publish high-impact papers within a short time frame both in print and online. As the field of trauma is a vast one, submissions in the form of Original Articles, Reviews, Case Reports, Short Communications, Technical Reports, Iatrogenic Trauma, Surgical Pathology and Letters-to-the-Editor are accepted. All submissions are reviewed by the relevant section editor and peer reviewers. In order to expedite the publication process we have expanded the number of our reviewers. Because publication time is an important factor for contributing authors accepted manuscripts are immediately slated for publication. There are no fees of any sort for authors or institutions and all articles are permanently available online free of charge. I would like to take the opportunity here to thank the journal team for their endeavors. I hope **TM** meets the high expectations of our readers and remains up to par in upholding international standards.

